# Questionnaires in clinical trials: guidelines for optimal design and administration

**DOI:** 10.1186/1745-6215-11-2

**Published:** 2010-01-11

**Authors:** Phil Edwards

**Affiliations:** 1 Department of Epidemiology and Population Health, London School of Hygiene and Tropical Medicine, London, UK

## Abstract

A good questionnaire design for a clinical trial will minimise bias and maximise precision in the estimates of treatment effect within budget. Attempts to collect more data than will be analysed may risk reducing recruitment (reducing power) and increasing losses to follow-up (possibly introducing bias). The mode of administration can also impact on the cost, quality and completeness of data collected. There is good evidence for design features that improve data completeness but further research is required to evaluate strategies in clinical trials. Theory-based guidelines for style, appearance, and layout of self-administered questionnaires have been proposed but require evaluation.

## Introduction

With fixed trial resources there will usually be a trade off between the number of participants that can be recruited into a trial and the quality and quantity of information that can be collected from each participant [1]. Although half a century ago there was little empirical evidence for optimal questionnaire design, Bradford Hill suggested that for every question asked of a study participant the investigator should be required to answer three himself, perhaps to encourage the investigator to keep the number of questions to a minimum [2].

To assess the empirical evidence for how questionnaire length and other design features might influence data completeness in a clinical trial, a systematic review of randomised controlled trials (RCTs) was conducted, and has recently been updated [3]. The strategies found to be effective in increasing response to postal and electronic questionnaires are summarised in the section on increasing data completeness below.

Clinical trial investigators have also relied on principles of questionnaire design that do not have an established empirical basis, but which are nonetheless considered to present 'good practice', based on expert opinion. The section on questionnaire development below includes some of that advice and presents general guidelines for questionnaire development which may help investigators who are about to design a questionnaire for a clinical trial.

As this paper concerns the collection of outcome data by questionnaire from trial participants (patients, carers, relatives or healthcare professionals) it begins by introducing the regulatory guidelines for data collection in clinical trials. It does not address the parallel (and equally important) needs of data management, cleaning, validation or processing required in the creation of the final clinical database.

### Regulatory guidelines

The International Conference on Harmonisation (ICH) of technical requirements for registration of pharmaceuticals for human use states:

'The collection of data and transfer of data from the investigator to the sponsor can take place through a variety of media, including paper case record forms, remote site monitoring systems, medical computer systems and electronic transfer. Whatever data capture instrument is used, the form and content of the information collected should be in full accordance with the protocol and should be established in advance of the conduct of the clinical trial. It should focus on the data necessary to implement the planned analysis, including the context information (such as timing assessments relative to dosing) necessary to confirm protocol compliance or identify important protocol deviations. 'Missing values' should be distinguishable from the 'value zero' or 'characteristic absent'...' [4].

This suggests that the choice of variables that are to be measured by the questionnaire (or case report form) is constrained by the trial protocol, but that the mode of data collection is not. The trial protocol is unlikely, however, to list all of the variables that may be required to evaluate the safety of the experimental treatment. The choice of variables to assess safety will depend on the possible consequences of treatment, on current knowledge of possible adverse effects of related treatments, and on the duration of the trial [5]. In drug trials there may be many possible reactions due to the pharmacodynamic properties of the drug. The Council for International Organisations of Medical Sciences (CIOMS) advises that:

'Safety data that cannot be categorized and succinctly collected in predefined data fields should be recorded in the comment section of the case report form when deemed important in the clinical judgement of the investigator' [5].

Safety data can therefore initially be captured on a questionnaire as text responses to open-ended questions that will subsequently be coded using a common adverse event dictionary, such as the Medical Dictionary for Drug Regulatory Activities (MEDRA). The coding of text responses should be performed by personnel who are blinded to treatment allocation. Both ICH and CIOMS warn against investigators collecting too much data that will not be analysed, potentially wasting time and resources, reducing the rate of recruitment, and increasing losses to follow-up.

Before questionnaire design begins, the trial protocol should be available at least in draft. This will state which outcomes are to be measured and which parameters are of interest (for example, percentage, mean, and so on). Preferably, a statistical analysis plan will also be available that makes explicit how each variable will be analysed, including how precisely each is to be measured and how each variable will be categorised in analysis. If these requirements are known in advance, the questionnaire can be designed in such a way that will reduce the need for data to be coded once questionnaires have been completed and returned.

### Questionnaire development

If a questionnaire has previously been used in similar trials to the one planned, its use will bring the added advantage that the results will be comparable and may be combined in a meta-analysis. However, if the mode of administration of the questionnaire will change (for example, questions developed for administration by personal interview are to be included in a self-administered questionnaire), the questionnaire should be piloted before it is used (see section on piloting below). To encourage the consistent reporting of serious adverse events across trials, the CIOMS Working Group has prepared an example of the format and content of a possible questionnaire [5].

If a new questionnaire is to be developed, testing will establish that it measures what is intended to be measured, and that it does so reliably. The validity of a questionnaire may be assessed in a reliability study that assesses the agreement (or correlation) between the outcome measured using the questionnaire with that measured using the 'gold standard'. However, this will not be possible if there is no recognised gold standard measurement for outcome. The reliability of a questionnaire may be assessed by quantifying the strength of agreement between the outcomes measured using the questionnaire on the same patients at different times. The methods for conducting studies of validity and reliability are covered in depth elsewhere [6]. If new questions are to be developed, the reading ease of the questions can be assessed using the Flesch reading ease score. This score assesses the number of words in sentences, and the number syllables in words. Higher Flesch reading scores indicate material that is easier to read [7].

### Types of questions

Open-ended questions offer participants a space into which they can answer by writing text. These can be used when there are a large number of possible answers and it is important to capture all of the detail in the information provided. If answers are not factual, open-ended questions might increase the burden on participants. The text responses will subsequently need to be reviewed by the investigator, who will (whilst remaining blind to treatment allocation) assign one or more codes that categorise the response (for example, applying an adverse event dictionary) before analysis. Participants will need sufficient space so that full and accurate information can be provided.

Closed-ended questions contain either mutually exclusive response options only, or must include a clear instruction that participants may select more than one response option (for example, 'tick all that apply'). There is some evidence that answers to closed questions are influenced by the values chosen by investigators for each response category offered and that respondents may avoid extreme categories [8]. Closed-ended questions where participants are asked to 'tick all that apply' can alternatively be presented as separate questions, each with a 'yes' or 'no' response option (this design may be suitable if the analysis planned will treat each response category as a binary variable).

Asking participants subsidiary questions (that is, 'branching off') depending on their answers to core questions will provide further detail about outcomes, but will increase questionnaire length and could make a questionnaire harder to follow. Similarly 'matrix' style questions (that is, multiple questions with common response option categories) might seem complicated to some participants, adding to the data collection burden [9].

### Style, appearance and layout

The way that a self-administered questionnaire looks is considered to be as important as the questions that are asked [9,10]. There is good evidence that in addition to the words that appear on the page (verbal language) the questionnaire communicates meaning and instructions to participants via symbols and graphical features (non-verbal language). The evidence from several RCTs of alternative question response styles and layouts suggests that participants view the middle (central) response option as the one that represents the midpoint of an outcome scale. Participants then expect response options to appear in an order of increasing or decreasing progression, beginning with the leftmost or uppermost category; and they expect response options that are closer to each other to also have values that are 'conceptually closer'. The order, spacing and grouping of response options are therefore important design features, as they will affect the quality of data provided on the questionnaire, and the time taken by participants to provide it [10].

Some attempts have been made to develop theory-based guidelines for self-administered questionnaire design [11]. Based on a review of psychological and sociological theories about graphic language, cognition, visual perception and motivation, five principles have been derived:

'Use the visual elements of brightness, colour, shape, and location in a consistent manner to define the desired navigational path for respondents to follow when answering the questionnaire;

When established format conventions are changed in the midst of a questionnaire use prominent visual guides to redirect respondents;

Place directions [instructions] where they are to be used and where they can be seen;

Present information in a manner that does not require respondents to connect information from separate locations in order to comprehend it;

Ask people to answer only one question at a time' [11].

Adherence to these principles may help to ensure that when participants complete a questionnaire they understand what is being asked, how to give their response, and which question to answer next. This will help participants to give all the information being sought and reduce the chances that they become confused or frustrated when completing the questionnaire. These principles require evaluation in RCTs.

Font size and colour may further affect the legibility of a questionnaire, which may also impact on data quality and completeness. Questionnaires for trials that enrol older participants may therefore require the use of a larger font (for example, 11 or 12 point minimum) than those for trials including younger participants. The legibility and comprehension of the questionnaire can be assessed during the pilot phase (see section on piloting below).

Perhaps most difficult to define are the factors that make a questionnaire more aesthetically pleasing to participants, and that may potentially increase compliance. The use of space, graphics, underlining, bold type, colour and shading, and other qualities of design may affect how participants react and engage with a questionnaire. Edward Tufte's advice for achieving graphical excellence [12] might be adapted to consider how to achieve excellence in questionnaire design, *viz*: ask the participant the simplest, clearest questions in the shortest time using the fewest words on the fewest pages; above all else ask only what you need to know.

Further research is therefore needed (as will be seen in the section on increasing data completeness) into the types of question and the aspects of style, appearance and layout of questionnaires that are effective in increasing data quality and completeness.

### Mode of administration

Self-administered questionnaires are usually cheaper to use as they require no investigator input other than that for their distribution. Mailed questionnaires require correct addresses to be available for each participant, and resources to cover the costs of delivery. Electronically distributed questionnaires require correct email addresses as well as access to computers and the internet. Mailed and electronically distributed questionnaires have the advantage that they give participants time to think about their responses to questions, but they may require assistance to be available for participants (for example, a telephone helpline).

As self-administered questionnaires have least investigator involvement they are less susceptible to information bias (for example, social desirability bias) and interviewer effects, but are more susceptible to item non-response [8]. Evidence from a systematic review of 57 studies comparing self-reported versus clinically verified compliance with treatment suggests that questionnaires and diaries may be more reliable than interviews [13].

In-person administration allows a rapport with participants to be developed, for example through eye contact, active listening and body language. It also allows interviewers to clarify questions and to check answers. Telephone administration may still provide the aural dimension (active listening) of an in-person interview. A possible disadvantage of telephone interviews is that participants may become distracted by other things going on around them, or decide to end the call [9].

A mixture of modes of administration may also be considered: for example, participant follow-up might commence with postal or email administration of the questionnaire, with subsequent telephone calls to non-respondents. The offer of an in-person interview may also be necessary, particularly if translation to a second language is required, or if participants are not sufficiently literate. Such approaches may risk introducing selection bias if participants in one treatment group are more or less likely than the other group to respond to one mode of administration used (for example, telephone follow-up in patients randomised to a new type of hearing aid) [14].

An advantage of electronic and web-based questionnaires is that they can be designed automatically to screen and filter participant responses. Movement from one question to the next can then appear seamless, reducing the data collection burden on participants who are only asked questions relevant to previous answers. Embedded algorithms can also check the internal consistency of participant responses so that data are internally valid when submitted, reducing the need for data queries to be resolved later. However, collection of data from participants using electronic means may discriminate against participants without access to a computer or the internet. Choice of mode of administration must therefore take into account its acceptability to participants and any potential for exclusion of eligible participants that may result.

### Piloting

Piloting is a process whereby new questionnaires are tested, revised and tested further before they are used in the main trial. It is an iterative process that usually begins by asking other researchers who have some knowledge and experience in a similar field to comment on the first draft of the questionnaire. Once the questionnaire has been revised, it can then be piloted in a non-expert group, such as among colleagues. A further revision of the questionnaire can be piloted with individuals who are representative of the population who will complete it in the main trial. In-depth 'cognitive interviewing' might also provide insights into how participants comprehend questions, process and recall information, and decide what answers to give [15]. Here participants are read each question and are either asked to 'think aloud' as they consider what their answer will be, or are asked further 'probing' questions by the interviewer.

For international multicentre trials it will be necessary to translate a questionnaire. Although a simple translation to, and translation back from the second language might be sufficient, further piloting and cognitive interviews may be required to identify and correct for any cultural differences in interpretation of the translated questionnaire. Translation into other languages may alter the layout and formatting of words on the page from the original design and so further redesign of the questionnaire may be required. If a questionnaire is to be developed for a clinical trial, sufficient resources are therefore required for its design, piloting and revision.

### Increasing data completeness

Loss to follow-up will reduce statistical power by reducing the effective sample size. Losses may also introduce bias if the trial treatment is an effect modifier for the association between outcome and participation at follow-up [16].

There may be exceptional circumstances for allowing participants to skip certain questions (for example, sensitive questions on sexual lifestyle) to ensure that the remainder of the questionnaire is still collected; the data that are provided may then be used to impute the values of variables that were not provided. Although the impact of missing outcome data and missing covariates on study results can be reduced through the use of multiple imputation techniques, no method of analysis can be expected to overcome them completely [17].

### Length

Longer and more demanding tasks might be expected to have fewer volunteers than shorter, easier tasks. The evidence from randomised trials of questionnaire length in a range of settings seems to support the notion that when it comes to questionnaire design 'shorter is better' [18]. Recent evidence that a longer questionnaire achieved the same high response proportion as that of a shorter alternative might cast doubt on the importance of the number of questions included in a questionnaire [19]. However, under closer scrutiny the results of this study (96.09% versus 96.74%) are compatible with an average 2% reduction in odds of response for each additional page added to the shorter version [18]. The main lesson seems to be that when the baseline response proportion is very high (for example, over 95%) then few interventions are likely to have effects large enough to increase it further.

There is a trade off between increased measurement error from using a simplified outcome scale and increased power from achieving measurement on a larger sample of participants (from fewer losses to follow-up). If a shorter version of an outcome scale provides measures of an outcome that are highly correlated with the longer version, then it will be more efficient for the trial to use the shorter version [1]. A moderate reduction to the length of a shorter questionnaire will be more effective in reducing losses to follow-up than a moderate change to the length of a longer questionnaire [18].

In studies that seek to collect information on many outcomes, questionnaire length will necessarily be determined by the number of items required from each participant. In very compliant populations there may be little lost by using a longer questionnaire. However, using a longer questionnaire to measure more outcomes may also increase the risk of false positive findings that result from multiple testing (for example, measuring 100 outcomes may produce 5 that are significantly associated with treatment by chance alone) [4,20].

### Other strategies to increase completeness

A recently updated Cochrane systematic review presents evidence from RCTs of methods to increase response to postal and electronic questionnaires in a range of health and non-health settings [3]. The review includes 481 trials that evaluated 110 different methods for increasing response to postal questionnaires and 32 trials that evaluated 27 methods for increasing response to electronic questionnaires. The trials evaluate aspects of questionnaire design, the introductory letter, packaging and methods of delivery that might influence the tendency for participants to open the envelope (or email) and to engage with its contents. A summary of the results follows.

### What participants are offered

#### Postal questionnaires

The evidence favours offering monetary incentives and suggests that money is more effective than other types of incentive (for example, tokens, lottery tickets, pens, and so on). The relationship between the amount of monetary incentive offered and questionnaire response is non-linear with diminishing marginal returns for each additional amount offered [21]. Unconditional incentives appear to be more effective, as are incentives offered with the first rather than a subsequent mailing. There is less evidence for the effects of offering the results of the study (when complete) or offering larger non-monetary incentives.

#### Electronic questionnaires

The evidence favours non-monetary incentives (for example, Amazon.com gift cards), immediate notification of lottery results, and offering study results. Less evidence exists for the effect of offering monetary rather than non-monetary incentives.

### How questionnaires look

#### Postal

The evidence favours using personalised materials, a handwritten address, and printing single sided rather than double sided. There is also evidence that inclusion of a participant's name in the salutation at the start of the cover letter increases response and that the addition of a handwritten signature on letters will further increase response [22]. There is less evidence for positive effects of using coloured or higher quality paper, identifying features (for example, identity number), study logos, brown envelopes, coloured ink, coloured letterhead, booklets, larger paper, larger fonts, pictures in the questionnaire, matrix style questions, or questions that require recall in order of time period.

#### Electronic

The evidence favours using a personalised approach, a picture in emails, a white background for emails, a simple header, and textual rather than a visual presentation of response categories. Response may be reduced when 'survey' is mentioned in the subject line. Less evidence exists for sending emails in text format or HTML, including a topic in email subject lines, or including a header in emails.

### How questionnaires are received or returned

#### Postal

The evidence favours sending questionnaires by first class or recorded delivery, using stamped return envelopes, and using several stamps. There is less evidence for effects of mailing soon after discharge from hospital, mailing or delivering on a Monday, sending to work addresses, using stamped outgoing envelopes (rather than franked), using commemorative or first class stamps on return envelopes, including a prepaid return envelope, using window or larger envelopes, or offering the option of response by internet.

### Methods and number of requests for participation

#### Postal

The evidence favours contacting participants before sending questionnaires, follow-up contact with non-responders, providing another copy of the questionnaire at follow-up and sending text message reminders rather than postcards. There is less evidence for effects of precontact by telephone rather than by mail, telephone follow-up rather than by mail, and follow-up within a month rather than later.

### Nature and style of questions included

#### Postal

The evidence favours placing more relevant questions and easier questions first, user friendly and more interesting or salient questionnaires, horizontal orientation of response options rather than vertical, factual questions only, and including a 'teaser'. Response may be reduced when sensitive questions are included or when a questionnaire for carers or relatives is included. There is less evidence for asking general questions or asking for demographic information first, using open-ended rather than closed questions, using open-ended questions first, including 'don't know' boxes, asking participants to 'circle answer' rather than 'tick box', presenting response options in increasing order, using a response scale with 5 levels rather than 10 levels, or including a supplemental questionnaire or a consent form.

#### Electronic

The evidence favours using a more interesting or salient e-questionnaire.

### Who sent the questionnaire

#### Postal

The evidence favours questionnaires that originate from a university rather than government department or commercial organisation. Less evidence exists for the effects of precontact by a medical researcher (compared to non-medical), letters signed by more senior or well known people, sending questionnaires in university-printed envelopes, questionnaires that originate from a doctor rather than a research group, names that are ethnically identifiable, or questionnaires that originate from male rather than female investigators.

#### Electronic

The evidence suggests that response is reduced when e-questionnaires are signed by male rather than female investigators. There is less evidence for the effectiveness of e-questionnaires originating from a university or when sent by more senior or well known people.

### What participants are told

#### Postal

The evidence favours assuring confidentiality and mentioning an obligation to respond in follow-up letters. Response may be reduced when endorsed by an 'eminent professional' and requesting participants to not remove ID codes. Less evidence exists for the effects of stating that others have responded, a choice to opt out of the study, providing instructions, giving a deadline, providing an estimate of completion time, requesting a telephone number, stating that participants will be contacted if they do not respond, requesting an explanation for non-participation, an appeal or plea, requesting a signature, stressing benefits to sponsor, participants or society, or assuring anonymity rather than participants being identifiable.

#### Electronic

The evidence favours stating that others have responded and giving a deadline. There is less evidence for the effect of an appeal (for example, 'request for help') in the subject line of an email.

So although uncertainty remains about whether some strategies increase data completeness there is sufficient evidence to produce some guidelines. Where there is a choice, a shorter questionnaire can reduce the size of the task and burden on respondents. Begin a questionnaire with the easier and most relevant questions, and make it user friendly and interesting for participants. A monetary incentive can be included as a little unexpected 'thank you for your time'. Participants are more likely to respond with advance warning (by letter, email or phone call in advance of being sent a questionnaire). This is a simple courtesy warning participants that they are soon to be given a task to do, and that they may need to set some time aside to complete it. The relevance and importance of participation in the trial can be emphasised by addressing participants by name, signing letters by hand, and using first class postage or recorded delivery. University sponsorship may add credibility, as might the assurance of confidentiality. Follow-up contact and reminders to non-responders are likely to be beneficial, but include another copy of the questionnaire to save participants having to remember where they put it, or if they have thrown it away.

The effects of some strategies to increase questionnaire response may differ when used in a clinical trial compared with a non-health setting. Around half of trials included in the Cochrane review were health related (patient groups, population health surveys and surveys of healthcare professionals). The other included trials were conducted among business professionals, consumers, and the general population. To assess whether the size of the effects of each strategy on questionnaire response differ in health settings will require a sufficiently sophisticated analysis that controls for covariates (for example, number of pages in the questionnaire, use of incentives, and so on). Unfortunately, these details are seldom included by investigators in the published reports [3].

However, a review of 15 RCTs of methods to increase response in healthcare professionals and patients found evidence for using some strategies (for example, shorter questionnaires and sending reminders) in the health-related setting [23]. There is also evidence that incentives do improve questionnaire response in clinical trials [24,25]. The offer of monetary incentives to participants for completion of a questionnaire may, however, be unacceptable to some ethics committees if they are deemed likely to exert pressure on individuals to participate [26]. Until further studies establish whether other strategies are also effective in the clinical trial setting, the results of the Cochrane review may be used as guidelines for improving data completeness. More discussion on the design and administration of questionnaires is available elsewhere [27].

### Risk factors for loss to follow-up

Irrespective of questionnaire design it is possible that some participants will not respond because: (a) they have never received the questionnaire or (b) they no longer wish to participate in the study. An analysis of the information collected at randomisation can be used to identify any factors (for example, gender, severity of condition) that are predictive of loss to follow-up [28]. Follow-up strategies can then be tailored for those participants most at risk of becoming lost (for example, additional incentives for 'at risk' participants). Interviews with a sample of responders and non-responders may also identify potential improvements to the questionnaire design, or to participant information. The need for improved questionnaire saliency, explanations of trial procedures, and stressing the importance of responding have all been identified using this method [29].

### Further research

Few clinical trials appear to have nested trials of methods that might increase the quality and quantity of the data collected by questionnaire, and of participation in trials more generally. Trials of alternative strategies that may increase the quality and quantity of data collected by questionnaire in clinical trials are needed. Reports of these trials must include details of the alternative instruments used (for example, number of items, number of pages, opportunity to save data electronically and resume completion at another time), mean or median time to completion of electronic questionnaires, material costs and the amount of staff time required. Data collection in clinical trials is costly, and so care is needed to design data collection instruments that will provide sufficiently reliable measures of outcomes whilst ensuring high levels of follow-up. Whether shorter 'quick and dirty' outcome measures (for example, a few simple questions) are better than more sophisticated questionnaires will require assessment of the costs in terms of their impact on bias, precision, trial completion time, and overall costs.

## Conclusion

A good questionnaire design for a clinical trial will minimise bias and maximise precision in the estimates of treatment effect within budget. Attempts to collect more data than will be analysed may risk reducing recruitment (reducing power) and increasing losses to follow-up (possibly introducing bias). Questionnaire design still does remain as much an art as a science, but the evidence base for improving the quality and completeness of data collection in clinical trials is growing.

## Competing interests

The author declares that he has no competing interests.
